# A Comparison of Pain Scores in Dysmenorrheic Patients With or Without Dyspeptic Symptoms

**DOI:** 10.7759/cureus.14437

**Published:** 2021-04-12

**Authors:** Yılmaz Sezgin, Asliddin Ahmedali

**Affiliations:** 1 Family Medicine, Istanbul Training Research Hospital, Istanbul, TUR; 2 Emergency Medicine, Nevşehir State Hospital, Nevşehir, TUR

**Keywords:** dysmenorrhea, dyspepsia, pain score

## Abstract

Introductıon: Dysmenorrhea attacks may be accompanied by extragenital symptoms such as nausea, vomiting, diarrhea, headache and leg pain and by emotional symptoms such as tension and irritability. Therefore, we think that dysmenorrheic symptoms may be more severe in patients with dyspeptic symptoms. The purpose of this study was to determine whether pain scores would differ between dysmenorrheic patients with or without dyspeptic symptoms.

Methods: Patients presenting to the emergency department with dysmenorrhea attacks and volunteering to participate were included in this case-control study. Subjects with dyspeptic symptoms were enrolled as the case group and those without dyspeptic symptoms were enrolled as the control group. Participants were administered the Faces Pain Scale and a questionnaire involving demographic characteristics.

Results: Pain scores on arrival were higher in the case group than in the control group (4.20 ± 0.71, 3.70 ± 0.74, n=30, p=0.011). A significant difference was observed between pain scores on arrival and at discharge in both the control and case groups. A decrease in pain scores was determined in all the subjects in the case group, while no change was observed in three volunteers in the control group.

Conclusions: We conclude that pain is significantly more severe in dysmenorrheic patients with dyspeptic symptoms.

## Introduction

Dysmenorrhea attacks may be accompanied by extragenital symptoms such as nausea, vomiting, diarrhea, headache and leg pain and by emotional symptoms such as tension and irritability [1]. Ischemia and myometrial contraction caused by prostaglandins of uterine origin are implicated in the pathology of dysmenorrhea [2]. Prostaglandin-suppressing non-steroid anti-inflammatory drugs are therefore the first choice in the pharmacological treatment of dysmenorrhea [3]. In addition to supporting treatments such as a fat-poor diet, vitamin B1, vitamin B6, vitamin E and magnesium, acupuncture and exercise have also been reported to reduce the symptoms of dysmenorrhea as alternatives to pharmacological treatment [4].

Increased activation of vagal afferents following either gastrointestinal dysrhythmia or dysmotility or following abnormal distension of the stomach, intestine or biliary tract also evokes nausea [5]. In addition, both vagus nerve and peripheral vagal nerve terminals play an active role in the development of symptoms of the gastrointestinal tract such as nausea, vomiting and diarrhea [6]. Some studies indicate that women experience more abdominal symptoms at the beginning of the follicular phase [7,8]. During menstruation, one-third of asymptomatic women may experience gastrointestinal symptoms and an increase in symptoms in almost 50% of women with irritable bowel syndrome [9-11].

Some studies have suggested that histamine released by histaminergic neurons in the fundus region of the rat stomach and in the pig ileum are involved in gastrointestinal symptoms [12,13]. Gastrin stimulates acid secretion by releasing histamine from enterochromaffin-like cells [14]. Moreover, vagal-induced acid secretion is also dependent on a histaminergic pathway but not on enterochromaffin-like cell histamine [15-18]. Recent studies have shown that histamine plays a role in small bowel and colon disorders such as irritable bowel syndrome and colitis [19,20].

There are studies suggesting that the uterus is stimulated by the innervation of the vagus nerve [21,22]. Moreover, it has been shown that the induction of pseudopregnancy in response to cervical stimulation was interrupted by abdominal vagotomy [23]. Additionally, histamine has been shown to cause contraction in the mouse uterus [24-26]. Therefore, we think that dysmenorrheic symptoms may be more severe in patients with dyspeptic symptoms. We also suggest that pain scores in dysmenorrheic patients with dyspeptic syndromes are higher than in patients without dyspeptic syndromes. The purpose of this study was to determine whether pain scores would differ between dysmenorrheic patients with or without dyspeptic symptoms.

## Materials and methods

This research was performed as a case-control study. This study was approved by the ethical committee. Thirty dysmenorrheic patients with dyspeptic symptoms presenting to the Nevşehir Public Hospital Emergency Department were enrolled as the case group, and 30 dysmenorrheic patients without dyspeptic symptoms were enrolled as the control group. Participants were administered a Face Pain Scale containing five different facial expressions with values between one and five at the time of the first presentation. The Face Pain Scale was repeated at the end of treatment. Participants were also administered a questionnaire involving demographic characteristics.

Patients of reproductive age, on the first day of the dysmenorrheic attack, with no history of any drug use, not using pregnancy-preventing drugs, not in the breastfeeding period and with no amenorrhea were included in the study.

All statistical analyses were performed with IBM SPSS Statistics (V25; IBM Corp., Armonk, NY). A P-value less than 0.05 was considered statistically significant. Demographic properties were compared with the chi-square test. Numeric data were expressed as mean values and were analyzed using the independent-samples t-test in independent groups and the paired-samples t-test in dependent groups.

## Results

No difference was observed between the case and control groups' demographic properties, in terms of age, body mass index (BMI), education level, marital status, smoking and alcohol use, chronic disease or medication use. A statistically significant difference was determined in terms of employment. Three patients (5%) of the case group regularly worked while 27 patients (45%) irregularly worked, while in the control group 14 patients (26.7%) regularly worked while 16 patients (23.3%) did not work (p=0.003; Table 1).

**Table 1:** Comparison of demographic properties between case and control groups. The statistically significant difference was accepted as p<0.05. ^a^Significant; p=0.003.

Characteristics of participants	Case (n=30)	Control (n=30)
Age (mean ± SD)	20.64±6.35	20.79 ± 2.87
BMI (mean ± SD)	21.87±2.95	21.67 ± 3.20
Education n (%)		
Prime level	7 (11.7)	3 (5)
High level	23 (38.3)	27 (45)
Employment n (%)^a^		
Regularly	3 (5)	14 (23.3)
Irregularly	27 (45)	19 (26.7)
Marital status n (%)		
Marriage	5 (8.3)	6 (10)
Single	25 (41.7)	24 (40)
Smoking n (%)		
Not use	23 (38.3)	20 (33.3)
Use	7 (11.7)	10 (16.7)
Alcohol n (%)		
Not use	30 (50)	27 (45)
Use	0 (0)	3 (5)
Chronic disease n (%)		
No	27 (45)	28 (46.7)
Yes	3 (5)	2 (3.3)
Medication n (%)		
Not use	29 (48.3)	28 (46.7)
Use	1 (1.7)	2 (3.3)

A statistically significant difference was also determined between the case and control groups in terms of mean pain scores. Pain scores at the time of presentation were significantly higher in the case group than in the control group (4.20 ± 0.71, 3.70 ± 0.74, n=30, p=0.011). Statistically significant differences between pain scores at the time of presentation and time of discharge were observed in both the case and control groups (Table 2).

**Table 2:** Comparison of pain scores at the time of presentation and discharge between two paired groups. *SD: standard deviation.

		n	Mean ± SD^*^	t-Value	p-Value
Case	Time of presentation	30	4.20 ± 0.71	19.33	0.001
	Time of discharge	30	1.86 ± 0.73		
Control	Time of presentation	30	3.70 ± 0.74	9.64	0.001
	Time of discharge	30	2.06 ± 0.82		

**Figure 1:**
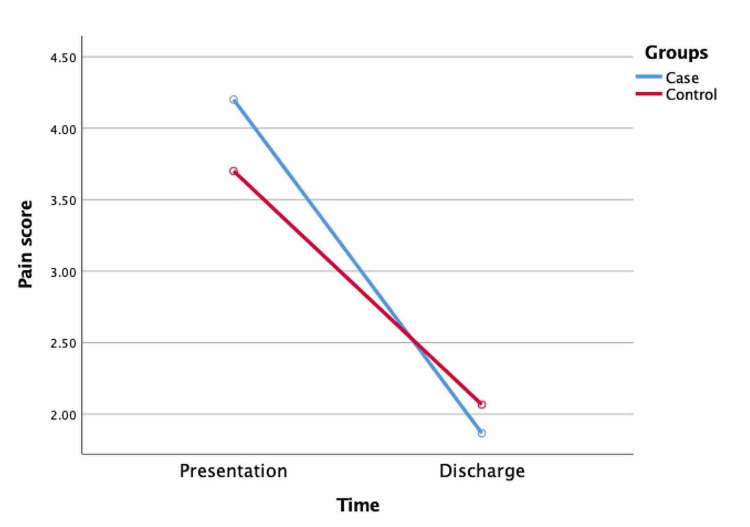
Comparison of pain scores at time of presentation and discharge between two independent groups.

A decrease in pain scores after discharge was determined in all the subjects in the case group, but no change in pain scores was observed in three volunteers in the control group. The decrease in pain scores between the time of presentation and time of discharge was greater in the case group than in the control group (2.33 ± 0.66, 1.63 ± 0.92, n=30, p=0.001; Figure 1).

## Discussion

We think that our study is unique since our scan of the literature revealed no previous such research. The absence of any difference between the control and case groups in terms of demographic characteristics other than employment shows that the study was well randomized and powerful.

Pain scores at the time of presentation to the emergency department in our study were significantly higher in the case group than in the control group. The reason for these pain scores being higher may be associated with dyspeptic symptoms. Although the major parasympathetic impulse system of the uterus derives from the sacral region of the medulla spinalis, the uterus also receives parasympathetic fibers via the vagus nerve [21,22]. Gastrointestinal symptoms being reported to vary during the phases of the menstrual cycle supports those studies suggesting that the uterus is under the effect of the vagus nerve [7,8,21-23]. Increased parasympathetic innervation gives rise to dysrhythmia, dysmotility, distension, nausea, vomiting and diarrhea in the gastrointestinal system and causes contractions in the uterus [5,6,21-23]. Symptoms seen in both the gastrointestinal system and the uterus in dysmenorrhea attack accompanied by dyspeptic symptoms resemble the effects of increased vagus nerve-mediated parasympathetic innervation. However, gastrointestinal symptoms being seen in only one-third of asymptomatic women during menstruation shows that a different mechanism similar to the parasympathetic effects of the vagus nerve is also involved [9-11].

Histamine possesses effects similar to increased parasympathetic innervation in both the uterus and the gastrointestinal system. Histamine release causes contraction in the uterus [24-26]. Gastrin has been shown to increase gastric acid secretion by causing histamine secretion from enterochromaffin-like cells while vagus nerve innervation causes an increase in gastric acid secretion by causing histamine secretion by a different histaminergic pathway [14-18]. In addition, the presence of histaminergic neurons has been demonstrated in both the fundus of the rat stomach and in the pig small bowel [12,13]. In the light of all this information, we think that gastrointestinal symptoms seen in some patients with dysmenorrhea derive from the release of histamine from vagus nerve-mediated histaminergic pathways or neurons. Moreover, studies both reporting that worsen symptoms of irritable bowel syndrome at the time of menstruation and indicating that histamine is involved in the irritable bowel syndrome support our thesis concerning histamine [9-11,19,20]. In other words, histamine may have played an effective role in dyspeptic symptoms seen during dysmenorrhea attacks.

In addition, the decrease in pain scores between the time of presentation and discharge was more pronounced in the case group. While pain scores decreased in all patients in the case group, no change was determined in three members of the control group. Being better improvement at discharge in the case group despite their pain scores being higher than those of the control group may be attributed to the addition of treatments aimed at the dyspeptic symptoms. The presence of a more pronounced improvement at the time of discharge in the case group compared to the control group may therefore derive from the H2 receptor antagonists added to treatment. We suggest that the increase in pain score at the time of arrival in the case group may be histamine-related. This finding from our study also supports our thesis concerning the effects of histamine in patients with dysmenorrhea. However, the fact that we do not know whether there exists in the uterus a pathway similar to the vagus nerve-mediated histaminergic pathways shown to be present in the gastrointestinal system weakens the validity of our thesis. For that reason, further research into this subject including animal experiments is needed regarding the parasympathetic innervation of the uterus.

## Conclusions

Consequently, severity of pain is worse in dysmenorrheic attacks accompanied by dyspeptic symptoms. We therefore think that the addition of an H2 receptor blocker drug to treatment during menstruation will contribute to a decrease in the symptoms of dysmenorrhea, a cause of considerable discomfort to women. We also think that wider-ranging studies are now needed on the subject.
